# Study protocol for a pragmatic randomised controlled trial of comparing enhanced acceptance and commitment therapy plus (+) added to usual aftercare versus usual aftercare only, in patients living with or beyond cancer: SUrvivors’ Rehabilitation Evaluation after CANcer (SURECAN) trial

**DOI:** 10.1186/s13063-024-08062-4

**Published:** 2024-04-02

**Authors:** Imran Khan, Stephanie J. C. Taylor, Clare Robinson, Elisavet Moschopoulou, Paul McCrone, Liam Bourke, Mohamed Thaha, Kamaldeep Bhui, Derek Rosario, Damien Ridge, Sheila Donovan, Ania Korszun, Paul Little, Adrienne Morgan, Olivier Quentin, Rebecca Roylance, Peter White, Trudie Chalder

**Affiliations:** 1 Barts and the London Centre for Primary Care, Wolfson Institute of Population Health, Queen Mary University of London, London, UK; 2 Barts and the London Pragmatic Clinical Trials Unit, Centre for Evaluation and Methods, Wolfson Institute of Population Health, Queen Mary University of London, London, UK; 3 Institute for Lifecourse Development, University of Greenwich, London, UK; 4 Dept. Allied Health Professionals, Sheffield Hallam University, Sheffield, UK; 5 Blizard Institute, Queen Mary University of London, London, UK; 6 Nuffield Department of Primary Care Health Sciences, Wadham College, University of Oxford, Oxford, UK; 7 The Academic Urology Unit, University of Sheffield, Sheffield, UK; 8 School of Social Sciences, University of Westminster, New Cavendish St, London, UK; 9 The Barts and the London Unit for Psychological Medicine, Centre for Psychiatry and Mental Health, Wolfson Institute of Population Health, Queen Mary University of London, London, UK; 10 Primary Care Research Centre, Faculty of Medicine, University of Southampton, Southampton, UK; 11 Independent Cancer Patient’s Voice (ICPV), 17 Woodbridge Street, London, UK; 12 University College London Hospitals NHS Foundation Trust, London, UK; 13 Institute of Psychiatry, Psychology & Neuroscience (IoPPN), King’s College London, DeCrespigny Park, London, UK

**Keywords:** Acceptance and Commitment Therapy, cancer survivor, Quality of Life, Pragmatic trial

## Abstract

**Background:**

Two million people in the UK are living with or beyond cancer and a third of them report poor quality of life (QoL) due to problems such as fatigue, fear of cancer recurrence, and concerns about returning to work. We aimed to develop and evaluate an intervention based on acceptance and commitment therapy (ACT), suited to address the concerns of cancer survivors and in improving their QoL. We also recognise the importance of exercise and vocational activity on QoL and therefore will integrate options for physical activity and return to work/vocational support, thus ACT Plus (+).

**Methods:**

We will conduct a multi-centre, pragmatic, theory driven, randomised controlled trial. We will assess whether ACT+ including usual aftercare (intervention) is more effective and cost-effective than usual aftercare alone (control). The primary outcome is QoL of participants living with or beyond cancer measured using the Functional Assessment of Cancer Therapy: General scale (FACT-G) at 52 weeks. We will recruit 344 participants identified from secondary care sites who have completed hospital-based treatment for cancer with curative intent, with low QoL (determined by the FACT-G) and randomise with an allocation ratio of 1:1 to the intervention or control. The intervention (ACT+) will be delivered by NHS Talking Therapies, specialist services, and cancer charities. The intervention consists of up to eight sessions at weekly or fortnightly intervals using different modalities of delivery to suit individual needs, i.e. face-to-face sessions, over the phone or skype.

**Discussion:**

To date, there have been no robust trials reporting both clinical and cost-effectiveness of an ACT based intervention for people with low QoL after curative cancer treatment in the UK. We will provide high quality evidence of the effectiveness and cost-effectiveness of adding ACT+ to usual aftercare provided by the NHS. If shown to be effective and cost-effective then commissioners, providers and cancer charities will know how to improve QoL in cancer survivors and their families.

**Trial registration:**

ISRCTN: ISRCTN67900293. Registered on 09 December 2019.

All items from the World Health Organization Trial Registration Data Set for this protocol can be found in Additional file 2 Table S1.

**Supplementary Information:**

The online version contains supplementary material available at 10.1186/s13063-024-08062-4.

## Introduction

### Background and rationale

Some two million people in the UK are living with or beyond cancer (also described as cancer ‘survivors’ and adopted for brevity in this paper) [1]. The number is increasing [2], with 50% of all those newly diagnosed now living for at least 10 years [3]. By 2028, an extra 55,000 people each year will survive for 5 years or more following their cancer diagnosis [4]. About a third have poor quality of life (QoL) and even more report other distress [5–7]. A national survey assessing the QoL of 3300 adult cancer survivors reported key issues or concerns including: fear of recurrence (57%), fatigue (43%), body image concerns (31%), and lack of exercise (30%) [5]. Poor QoL is also associated with unemployment in those of working age [8], with up to a third losing their work after cancer diagnosis [5]. The NHS Cancer Quality of Life survey in April 2023 reported that amongst over 100,000 patients who have had their cancer diagnosis for longer than 18 months, 80% across all age groups report a problem with an aspect of their health. This includes anxiety and depression (52%), pain and discomfort (64%), and problems with usual activities (52%) [9]. There is wide variation in NHS ‘aftercare’ [6, 10], and interventions to improve QoL and address the unmet needs of patients are only moderately effective, and often unavailable [10, 11].

Two key policy documents have highlighted the importance of cancer survivorship [6, 7]; the National Cancer Survivorship Initiative included goals to reduce the proportion of people with unmet physical and psychological support needs and to increase the proportion of cancer survivors able to work [6]. The report recommended self-management, after appropriate assessment and treatment, and both physical activity programmes and vocational support [6]. The goals of the Independent Cancer Taskforce, established by NHS England, included every person with cancer having access to a ‘recovery package’ of aftercare with ‘stratified pathways of follow-up care (7)’. Some recommendations focused on the need for more research into survivorship issues and QoL, including return to work [7]. Other recommendations covered the need for rehabilitation services and specific treatment for depression [7]. The need for evidence-based interventions to facilitate a return to a normal life in those living with and beyond cancer is also increasingly being recognised by the professions [12, 13].

In attempting to address the problems that cancer survivors face, non-pharmacological interventions such as cognitive behavioural therapy (CBT), exercise, and mindfulness-based stress reduction (MBSR) have shown to be effective at improving overall QoL, mostly in the short term [11]. In addition, only exercise and CBT consistently showed efficacy, although the effect sizes were small to moderate, with limited long-term follow-up [11]. A recent systematic review of an ACT intervention (and interventions based on the principles of ACT) in adult cancer survivors highlights ACT as an effective intervention to address the issues concerning cancer survivors but recommended more robust studies [14]. In a trial using ACT in 135 cancer survivors, the intervention was delivered in group sessions by community social workers trained in ACT. The trial demonstrated accelerated psychological recovery and energy levels with the intervention [15]. There have also been a few small trials in cancer patients who were in active treatment [16], but no large trials of ACT in cancer survivors looking at QoL, where the intervention is delivered to participants one-to-one by trained therapists [11, 14].

Acceptance and commitment therapy (ACT) lends itself to addressing the aforementioned problems in cancer survivors. ACT is a ‘third wave’ psychological intervention that was purportedly developed following questions some researchers had over the validity and effectiveness of cognitive restructuring in cognitive behavioural therapy (CBT) versus behavioural interventions, for the treatment of depression [17]. CBT identifies thoughts that cause distress, and alternative perspectives are then explored if and when appropriate [16]. In contrast to CBT, third-wave approaches (like ACT) are not concerned with challenging the content/frequency of negative thoughts and emotions. ACT aims to increase psychological flexibility, which in this context, refers to the ability to adapt to demands, shift perspectives, and balance competing desires and needs through processes involving acceptance, mindfulness, commitment, and values-based behaviour change [14].

Overall, an ACT intervention which is person-centred, and integrated with both an exercise intervention [18] and work support—when appropriate to the individual’s life goals [19], is therefore appropriate in addressing the needs of those living with and beyond cancer [16, 20]. As we are integrating ACT with options to support work/vocational activity and exercise, we are therefore calling this integrated approach ‘ACT Plus (+)’. An evaluation of the effectiveness, and economic outcomes of an intervention found to be promising in cancer survivors, would be an important contribution to the NHS [10, 11]

Here, we describe how we will evaluate the effectiveness and cost-effectiveness of our ACT+ intervention.

### Aims and objectives

The aim is to evaluate whether ACT+ in addition to usual aftercare is more effective and cost-effective than usual aftercare alone in improving QoL in people living with and beyond cancer and experiencing low QoL. The specific objectives are as follows:

- To conduct a randomised, controlled trial to examine the effectiveness of ACT+ in addition to usual care compared to usual care alone on clinical outcomes at 52-week follow-up
- To determine the cost-effectiveness of ACT+ with usual care versus usual care alone in terms of quality-adjusted life years (QALYs)
- To determine the mechanism of treatment, via the investigation of hypothesised mediators
- To determine variations in treatment effect by participant characteristics, via the exploration of potential moderators

### Trial design

This is a multi-centre, parallel group, theoretically driven, pragmatic, randomised controlled, superiority trial including an internal pilot. We will also undertake a health economic evaluation and a process evaluation with a formal investigation of hypothesised mediators. The primary outcome will be the Functional Assessment of Cancer Therapy: General scale (FACT-G) at 52-week follow-up. The unit of randomisation is the individual study participant performed as block randomisation with a 1:1 allocation ratio. Patients living with cancer and carers including those from an ethnically diverse background are involved in the design and implementation of the trial to improve the relevance and overall quality of the research.

### Methods: participants, interventions, and outcomes

#### Study setting and participants

Participants will be recruited from secondary care settings within five London NHS Trusts and one NHS Trust in Sheffield, specifically from the cancer follow-up clinics associated with our cancer groups of interest.

The ACT+ intervention will be delivered by participating therapists from NHS Talking Therapies (formerly known as Improving Access to Psychological Therapies, IAPT), specialist psychological therapy services, or cancer charities providing counselling or psychological therapies. NHS Talking Therapy sites are selected based upon their geographical proximity to the patient populations attending the recruiting sites.

The full list of recruitment and intervention sites is available from the authors.

#### Eligibility criteria

Eligible participants must have attended, or are being followed up by, a participating cancer clinic and must be within 24 months of having completed cancer treatment with curative intent or be in long term remission (where applicable). Eligible participants must also be considered to have low QoL as determined by a score of 78 or less (out of a maximum score of 108 and a minimum of 0) on the Functional Assessment of Cancer Therapy – General (FACT-G) [21, 22]. A full list of inclusion and exclusion criteria are provided in Table 1.

**Table 1:** Inclusion and exclusion criteria

Inclusion criteria	Exclusion criteria
Patients within 24 months of having completed cancer treatment of the index cancer (or about to complete) with curative intent/long term remission for: breast cancer, lower gastrointestinal cancer, a urological cancer, a haematological cancer, head and neck cancer, and any other common cancer with good survival Aged 18 years or over Ability to give informed consent Sufficient fluency in spoken English to be able to participate in a talking-based therapy delivered in English With a score of 78 or less on the Functional Assessment of Cancer Therapy – General (FACT-G)	Will not have completed their cancer treatment by the commencement of the trial (excepting those receiving long-term, ongoing maintenance treatment, e.g. androgen suppression therapy in prostate cancer) Receiving treatment for symptom control alone Currently receiving another psychological intervention (NB participants taking. antidepressants or anxiolytic drugs remain eligible) Other serious co-morbid condition which would make it difficult for the participant to receive a talking-based one-to-one intervention Require urgent psychiatric or clinical psychology assessment

#### Recruitment

Identification of potentially eligible participants will be carried out by the site NHS clinical team and study research assistants. Interested potential participants will be asked three screening questions. This is done using a 5-point Likert scale, where a score of 1 is poor, and 5 is excellent. The three questions are as follows: How would you rate your physical health? How would you rate your feelings of wellbeing? How would you rate your quality of life? For those whose score indicates they may require support (a score of 10 or lower) a member of the research team will, with their permission, administer the FACT-G. Those participants who score 78 or less on the FACT-G and remain interested in the study will be sent the baseline questionnaire along with the informed consent form by a study research assistant. Participants will need to return both or complete them online using a secure data entry system, before joining the study. On return of the baseline questionnaire, the research assistant will check to see if the participant remains eligible on the FACT–G score criterion. Thus, only participants whose FACT-G QoL scores have been below 79 on two separate occasions, typically separated by about a week, will be eligible to join the study, and randomised to either receive the intervention or usual care only.

#### Additional consent provisions for collection and use of participant data and biological specimens

Participants will have the option on the ICF to give their consent to be approached for participation in a separate interview study. The protocol for the interview study can be found: https://openresearch.nihr.ac.uk/articles/3-24. Participants also provide their consent to be included in a 2-year follow-up if they have been in the study for 2 years by the time of last participant, last 52-week follow-up.

#### Intervention

ACT is an intervention that aims to increase psychological flexibility. Psychological flexibility refers to an individual’s ability to adapt to demands, shift perspectives, and balance competing desires and needs [16, 23–25]. This means that ACT could help a patient to accept what cannot be changed (e.g. that the cancer might recur), while committing themselves to the things they can change (i.e. meeting their goals in life, irrespective of having had cancer).

We developed the intervention to be integrated with options for work/vocational activity support and exercise in ways that are tailored to each participant’s personal values and goals, and therefore called this ‘ACT Plus (+)’. We also developed a participant handbook and manuals for therapists. The process of developing and refining the ACT+ intervention will be reported separately elsewhere (in preparation).

The intervention is delivered by therapists who had undergone specifically designed training in ACT+ led by the senior study therapist (co-chief investigator TC). Full details of the SURECAN ACT+ training and its evaluation have been published [26]. The core professions of the therapists include clinical psychologists, high intensity therapists (from NHS Talking Therapies), cognitive behaviour therapists trained in ACT, and trained counsellors. Treatment integrity will be assessed at the end of the trial guided by Perepletchikova et al.’s treatment integrity procedures checklist [27].

The sessions can be delivered face-to-face (at the therapist’s main practice), by phone or via online video calls, according to participant preference. The intervention involves up to eight sessions delivered at weekly or fortnightly intervals. Sessions last around 1 h and include homework to be completed prior to the next session. All sessions should take place within a range of 14–20 weeks from a participant being allocated to the intervention arm at randomisation (depending on the frequency of sessions, i.e. weekly or fortnightly, and allowing occasions for missed/rescheduled sessions). Participants are also provided with the ACT+ participant handbook. Eligible participants randomised to the intervention arm will receive the ACT+ intervention in addition to usual aftercare.

#### Explanation for choice of comparators

Since we wish to know whether the addition of ACT+ is effective and cost-effective compared to usual aftercare, it is necessary to make the comparison arm treatment as usually provided by the NHS. Allowing a usual care control arm also solves the problem of offering a standardised usual aftercare intervention, which would likely duplicate what patients already receive (see below). However, we will enhance the control by providing the Macmillan Cancer Support leaflet, signposting the participant to aftercare available.

Usual after-care varies across different cancer groups even within the same trust as our development work demonstrated [10]. A national survey of oncology health care practitioners, undertaken as part of our programme development work in 2015 (prior to the COVID-19 pandemic), showed that specific interventions that were offered by the NHS included the following: dietary advice (72%), a medical assessment (69%), exercise advice (65%), a one off ‘end of care’ assessment (62%), and counselling (61%). Interventions such as CBT (16%), mindfulness (21%), and return to work support (20%) were infrequently offered [10].

#### Criteria for discontinuing or modifying allocated interventions

Intervention arm participants may discontinue receiving the intervention at any point they request.

#### Strategies to improve adherence to interventions and assessment of compliance

The therapists undergo monthly drop-in supervision sessions which are conducted by TC. The supervision sessions promote quality of therapy and adherence to the study therapy and protocol. Individual advice and support for therapists is also available as required.

#### Outcomes

##### The primary outcome

The primary outcome will be the total score of the Functional Assessment of Cancer Therapy: General scale (FACT-G) at 52 weeks follow-up; FACT-G is a generic measure of physical, social, emotional, and functional QoL; it ranges from 0 to 108 with a higher score indicating a better QoL [21]. The method of aggregation will be the mean FACT-G score at 52 weeks for both arms. In the programme grant development work comparing different quality of life measures related to cancer, the FACT-G was found to address their concerns and was the easiest to complete [28].

##### Secondary outcomes

The secondary outcomes and mediator measures shown below (unless specified) will be reported at all timepoints: baseline, 7 weeks, 16 weeks, 52 weeks, and 2 years (where applicable). The method of aggregation will be mean scores for both arms. Further details will be presented in the forthcoming statistical analysis plan.

1. The total score of the FACT-G at all other timepoints
2. The FACT-G sub-scale total scores (physical well-being [range 0–28], social/family well-being [range 0–28], emotional well-being [range 0–24] and functional well-being [range 0–28]. With higher scores indicating better QoL
3. Fear of cancer recurrence [29]. Four question self-report scale
4. The Impact of Cancer scale (IOCv2): positive and negative impacts subscales [30]
5. Anxiety and depression, measured using the Hospital Anxiety and Depression Scale (HADS) [31]
6. The Chalder Fatigue Scale (CFQ) [32] which is composed of 11 items, each of which is answered using a 4-point Likert scale ranging from 0 (less than usual) to 3 (much more than usual). The maximum score is therefore 33, and a higher score is associated with a worse health state
7. Measuring independent physical activity (frequency of occurrences, and duration in minutes) using an modified version of the Godin leisure score index questionnaire [33]
8. The EuroQol (EQ-5D-5L) [34] will be used to measure health-related QoL and to derive quality-adjusted life years (QALYs). The EQ-5D-5L consists of five domains (mobility, usual activities, self-care, pain/discomfort, and anxiety/depression). Each domain is scored between 1 (no problems) and 5 (extreme problems)
9. Health and social care utilisation and use of informal care measured using an adapted version of the Client Service Receipt Inventory [35]. This will cover the three months before randomisation and the periods up to 16-week and 52-week follow-up. The CSRI will be self-completed by participants. We will also collect employment status
10. Engagement and frequency of engagement in any new meaningful activities (e.g. new work, job, hobbies, or interests) at 16- and 52-week follow-up. This will be analysed as a categorical variable and therefore represented as a descriptive table, for each arm

### A priori mediator measures, at 7 weeks

1. Acceptance and Action Questionnaire (AAQ-II): the AAQ measures psychological flexibility using a 7-item scale [36]
2. Values Questionnaire (VQ): 10-item self-report measure assessing the extent to which one lives consistently with their values [37]
3. Committed Action Questionnaire (CAQ): the CAQ 8-item measure is derived from an original scale of 24 items [38]. Committed action is goal-directed, flexible persistence
4. Beliefs about emotions scale (BAE): this 12-item scale represent types of beliefs about the unacceptability of experiencing and expressing emotions that have been specified in cognitive models [39]

### Participant timeline

**Fig. 1:**
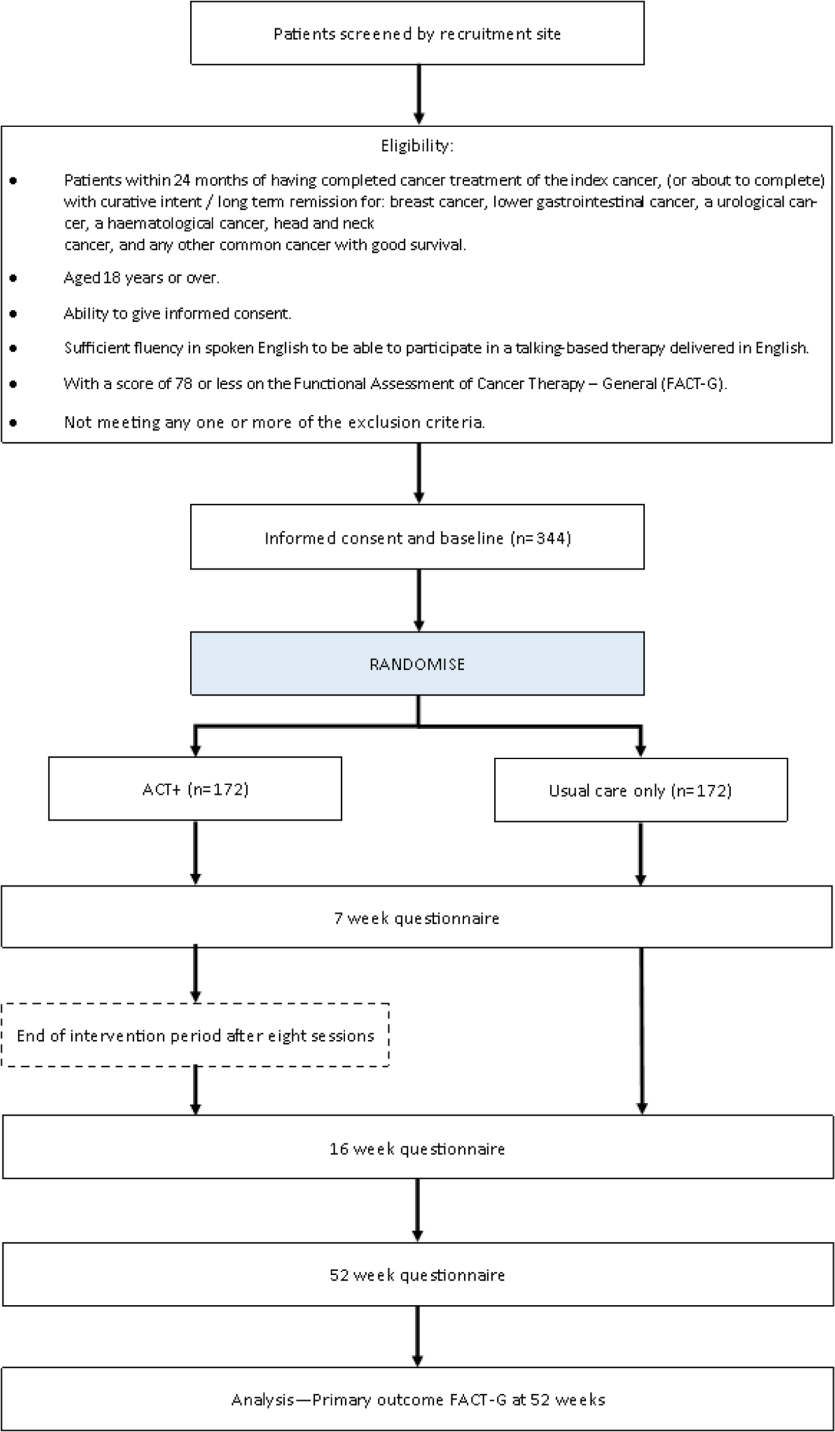
Participant flow

The participant timeline is shown in Fig. 1.

### Sample size

A total sample size of 266 (133 per arm) would provide 90% power to detect a minimally clinically important effect size (Cohen’s *D*) of 0.4 assuming a 2-sided 5% significance level. The effect size is based on existing literature, determined previously as 0.42 and 0.46 for FACT-G [40, 41]. This effect size represents a difference of 6 points on FACT-G in a cancer population [22]. Assuming each therapist sees 10 participants, an intracluster correlation coefficient (ICC) of 0.01, and allowing for drop-out by 52 weeks of 15%, the estimated total sample size required is 344 (172 per arm) patients to be recruited over 33 months. This represents an average of 10.4 participants per month in total across participating sites. Based on the numbers of patients seen in three sites (845 per annum), this sample size can be achieved assuming a recruitment rate of at least 20% of eligible participants. The sample size assuming independent observations was calculated using the power command in Stata. This was then manually multiplied by the design effect and divided by the proportion expected at follow-up to account for loss to follow-up.

Average estimates of therapist effects for RCTs have been shown to be 0.08, however they are highly variable across studies due to their dependence on the specific outcomes, research design and statistical analysis used [42, 43]. ACT+ uses a manualised approach with structured intervention sessions, and therapists receive training and monthly supervision on its delivery. End of treatment is scheduled for 16 weeks, while the primary outcome of FACT-G score is collected at 52 weeks. These features are likely to reduce therapist effects to negligible levels at the point of primary outcome collection. However, to be conservative we have allowed for the presence of low levels of clustering, estimated at 0.01.

### Methods: assignment of interventions

#### Randomisation

After confirmation of eligibility and collection of informed consent and baseline measures, participants will be randomised to either the intervention or usual aftercare, 1:1. The randomisation will be stratified by broad cancer type (breast, lower gastro-intestinal, urological, haematological, head and neck) and centre (*n* = 6) using different sized randomly permuted blocks. To maintain blinding, the allocation sequence will be generated by a statistician independent to the trial using the ralloc command in stata [44] overseen by the Pragmatic Clinical Trials Unit at Queen Mary (https://www.qmul.ac.uk/pctu/).

#### Concealment mechanism

To maintain allocation concealment, the block sizes used in the sequence generation are unknown to those recruiting participants, and the full randomisation lists are not available to any member of the research team. The randomisation list was integrated into a central REDCap randomisation database which is accessed via the internet to perform a randomisation. Upon entering a participant’s details, the system returns the appropriate allocation in the sequence for that individual.

#### Who will be blinded

Research assistants collecting any outcome data by phone will be blinded to participants’ allocated arm and trial statisticians will be blinded to allocation until finalisation of the statistical analysis plan. The trial manager and the research administrator are the only members of the study team who will be unblinded to ensure that participants randomised to the intervention receive the intervention in a timely fashion.

#### Implementation

The trial manager who is unblinded will receive notifications to randomise eligible participants by the research assistants. Participant details are entered onto the REDCap database once their eligibility has been confirmed.

### Methods: data collection, management, and analysis

#### Plans for assessment and collection of outcomes

The complete list of outcomes is listed in the ‘outcomes’ section above and listed in Fig. 3. Before commencing recruitment, each site receives training on the study design, protocol procedures (via site initiation visit), and data collection procedures (REDCap database training). All members of the site research team are expected to be appropriately trained and work in accordance with GCP guidelines, which is documented in the trial master file (TMF) and investigator site file (ISF).

At baseline, 7 weeks (approximately mid therapy), 16 weeks (approximately end of therapy), and 52 weeks following randomisation, participants will be asked to complete questionnaires related to the outcome measures. Further outcome assessment data will be collected at 2 years after randomisation for participants for whom, before the last recruited participant’s 52-week follow-up time point, would have had 2 years elapse from randomisation. All outcome measures will be collected by either direct participant input into a secure online study-developed database (developed by the QMUL Pragmatic Clinical Trials Unit) or completed via paper questionnaires and returned for data entry by the study team. The questionnaires will not be available in any languages other than English.

#### Health economics

For the economic evaluation, we will combine health and social care costs with the primary outcome measure and quality-adjusted life years (QALYs). Costs will be calculated for the intervention based on therapist time required, and other costs will be derived from the CSRI combined with recognised unit costs. For a societal perspective, informal care and lost employment costs will be valued using average wage rates. QALYs will be estimated using the EQ-5D-5L combined with UK tariffs and using area under the curve methods [35]. Cost differences will be compared between the groups across the whole follow-up period using a regression model with adjustment made for baseline costs. QALY differences will adjust for baseline EQ-5D-5L. Cost-effectiveness will be assessed from a health and social care perspective. If the intervention results in higher costs and better outcomes, then we will divide incremental costs (intervention minus usual care) by incremental QALY to generate an incremental cost-effectiveness ratio. Uncertainty around the cost-effectiveness results will be explored using cost-effectiveness planes and acceptability curves from 1000 cost-outcome combinations from bootstrapped regression models. The cost per unit improvement on the FACT-G will also be calculated. To assess the long-term cost-effectiveness of ACT+, we will develop a decision model using Markov processes. This will entail defining health states over time to which costs and QALYs will be attached. The structure of the model will be developed within the early stages of the programme and will consist of states defined both by clinical severity and quality of life. Costs and probabilities for the model will be derived from published literature, the trial, and expert opinion. The model will be subjected to extensive deterministic and probabilistic sensitivity analyses.

#### Meditators and moderators

**Fig. 2:**
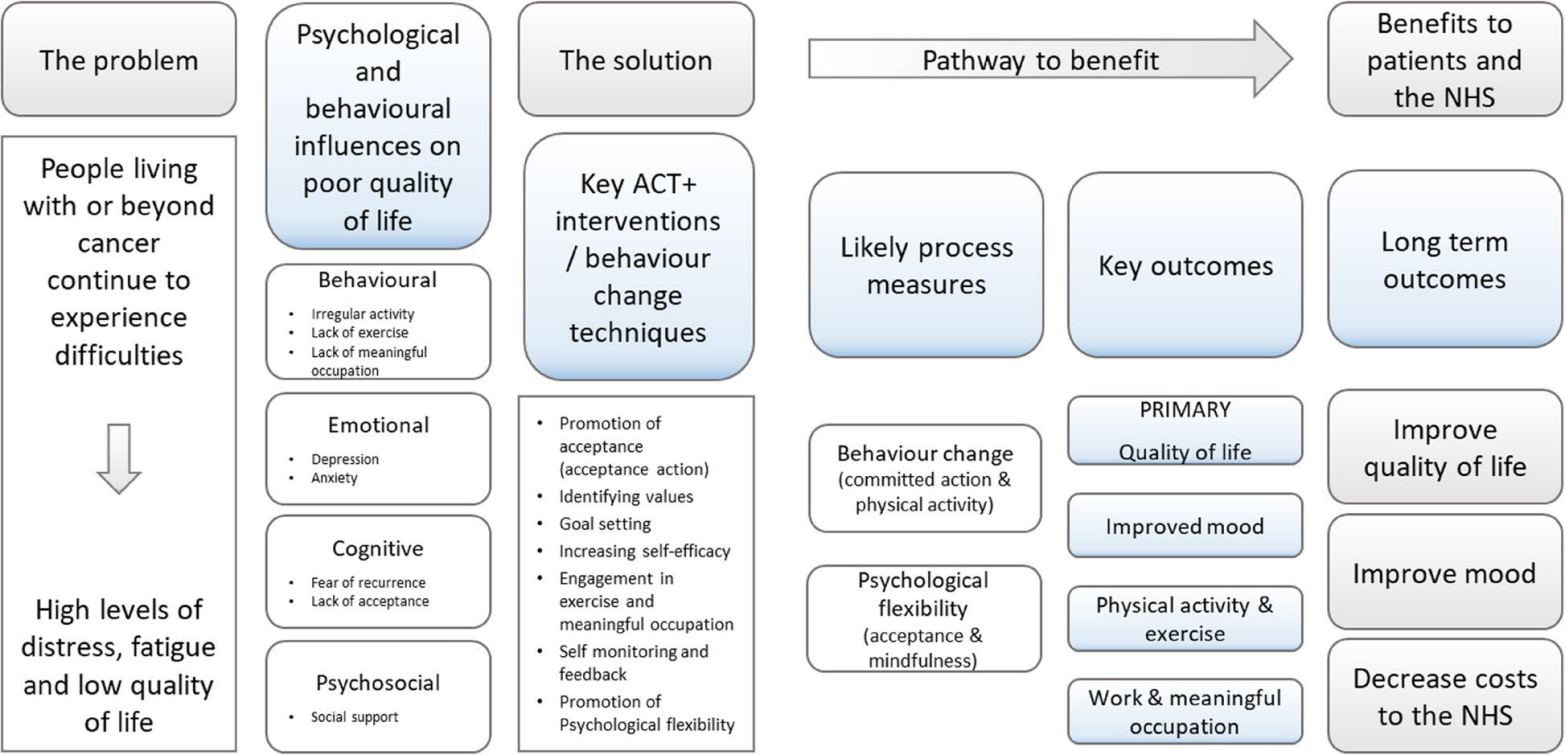
Logic model for the ACT+ intervention

Mediation analysis will investigate whether the mechanisms hypothesised to bring about improvements in outcomes after ACT+ (see Fig. 2) can be shown to operate on the primary outcome, QoL as measured by the FACT-G scale, and the secondary outcome of fatigue as measured by the Chalder Fatigue Scale at both 16 and 52. The mediators assessed at 7 weeks (mid-treatment) are commensurate with the theory of ACT+. Mediator analyses will be reported separately following the AGReMA reporting guideline [45].

Moderators will include age, gender, type of cancer, severity of depression, and the effects of loneliness and worry in relation to COVID-19 at baseline.

#### Adverse events

**Fig. 3:**
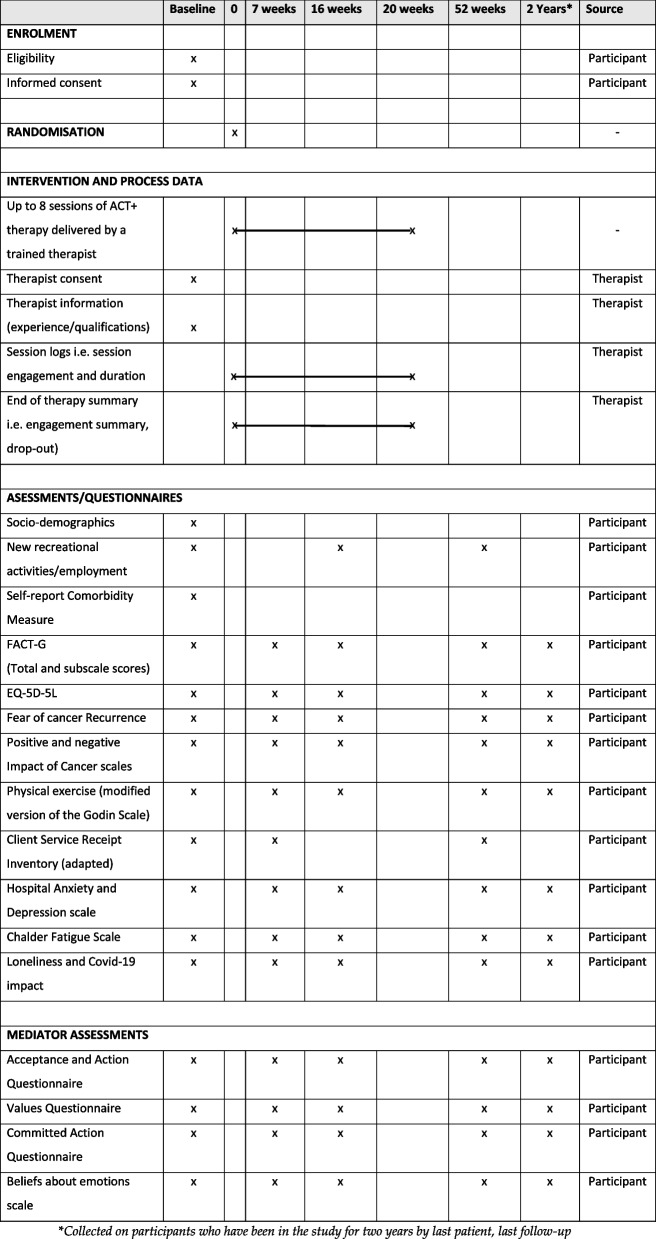
Schedule of enrolment, interventions, and assessments

All adverse events (AE) and severe adverse events (SAE) as defined (see the ‘Adverse event reporting and harm’ section) will be reported in the trial analysis by arm. In addition, we will report the proportion of patients who deteriorate on the FACT-G and HADS at 52 weeks from baseline, where at least four ACT+ sessions (50%) were delivered (Figs. 2 and 3) [46].

#### Plans to promote participant retention and complete follow-up

Participants are sent the questionnaires either by post or via an online survey, according to participant preference. When posting the questionnaire, researchers will also send the participant a self-addressed envelope, a pen, and an unconditional gift voucher of £5 at all time-points. The consent form will only be posted at baseline. Participants who complete the questionnaire online will be sent a link to complete the questionnaire via email. Non-responders at each time point will be telephoned to check whether they have received the questionnaire, and automated reminders are sent for online questionnaires. If it is established that the participant is unable or unwilling to fully complete the questionnaires, the study team will offer the participant the option of completing only the FACT-G, the EQ-5D-5L, and the CSRI, as they are needed to evaluate the primary outcomes (effectiveness and health economic) and cost per QALY. A primary outcome CRF has been developed for this purpose. Researchers will also offer participants the opportunity to complete these outcomes over the telephone when appropriate.

#### Data management

The SURECAN study team will work closely with staff at participating sites to ensure accurate (complete, valid, and reliable) collection of data. Completeness, range, and consistency checks will further enhance the quality of the data. Two levels of data validation will be incorporated when entering data (either online via direct participant data entry or entered directly onto the REDCap database by the study team). The first level will aim to prevent obviously invalid data from being entered, e.g. entering a date of birth that occurred after the date of consent. The second level checks for data completeness include any unusual data entered, i.e. a variable that was outside of the pre-defined range. The site PI is responsible for ensuring that all data queries are resolved. Ongoing data entry, validation at adherence to the trial protocol at sites will be closely monitored by the study team, and any concerns will be raised to the participating sites.

All PCTU SOPs with regard to data management will be adhered to by the study team. A data management plan will be written to cover all aspects of managing the data.

#### Confidentiality

Information related to participants will be managed in accordance with General Data Protection Regulation (GDPR), NHS Caldecott Principles, The Research Governance Framework for Health and Social Care, and the conditions of Research Ethics Committee Approval.

The participant information sheets will set out arrangements relating to confidentiality, security, storage of data, and accessibility of data only to the study team. All paper documentation containing identifiable participant data such as consent forms and CRFs will be kept in locked filing cabinets at the NHS sites or at QMUL offices. All participants will be assigned a unique SURECAN participant ID. The CRFs will be pseudo-anonymised with the participant ID. The study team will keep logs that will contain personal information collected on participants to facilitate the running of the trial. These logs will be kept on a secure, password-protected shared drive, only accessible by appropriate study team members with the password. At participating sites, identifiable information of participants will be kept on secure NHS trust computers only accessible by site staff.

Data collected using REDCap will be pseudonymised by using the unique study participant ID. REDCap data is stored securely on the secure virtualised environment at the Barts Cancer Centre (BCC). The BCC environment requires dual factor authentication to access the portal and the folders where the data are stored are only accessible to the appropriate members of the PCTU and the SURECAN study team.

The audio recorded intervention session data will be collected using an encrypted audio recording device. Once the data has been collected, it will be stored on an encrypted USB and deleted from the audio recording device. Sessions recorded using the online video calling platform are saved directly onto the encrypted USB. The encrypted USBs will be securely returned to the study team.

### Statistical methods

#### Statistical methods for primary and secondary outcomes

Prior to any analysis taking place, a statistical analysis plan will be developed and made publicly available, containing a more detailed and technical description of the analysis to be performed. To ensure transparency and reproducibility, its content will follow the guidelines proposed by Gamble et al. [47].

The analysis will be reported in line with the CONSORT guidelines and the extension for non-pharmacologic treatment interventions [48]. Baseline demographic and clinical outcomes will be summarised by treatment group. Normally distributed data will be summarised by mean (standard deviation); non-normally distributed data will be presented as median (interquartile range) and categorical variables presented as *n* (%).

The analysis will follow intention-to-treat principles, where participants with available data are included in the treatment group to which they were randomised. Hypothesis tests will be two-sided and estimated treatment effects will be accompanied by a 95% confidence interval. For all analyses, a significance level of 5% will be used.

The primary analysis will be a mixed effects linear regression of the total FACT-G score at 52 weeks with a random effect for therapist, fixed effects for baseline score, and randomisation stratification factors. Secondary outcomes will be modelled similarly.

To explore the treatment effects over time, the analysis will be repeated with multilevel mixed effects models with a nested structure to include an additional random effect for individual, a fixed effect for time, and an interaction between treatment and time. Safety data will be summarised by treatment group.

Predictors of costs and cost-effectiveness will also be identified, the latter being calculated as the monetary value of QALYs minus therapy costs. Generalised or standard linear models will be used as appropriate.

#### Interim analyses

No formal interim analyses comparing outcome data between the two groups is planned.

#### Methods for additional analyses (e.g. subgroup analyses)

The primary analysis will be repeated to include an interaction term for the treatment effect and potential moderators (subgroups/moderators). Subgroups will include factors such as age, gender, type of cancer, severity of depression, and the effects loneliness and worry in relation to COVID-19 at baseline. Full details of additional analyses will be included in an analysis plan.

#### Methods in analysis to handle protocol non-adherence and any statistical methods to handle missing data

Sensitivity analyses of the primary analysis will be conducted (i) on the subsample who adhere to the protocol (ii) to explore the dose-response relationship between intervention attendance and outcome and (iii) to assess the robustness of the primary conclusions to the missing data assumptions. Multiple imputation will be undertaken, provided we have strong predictors of missingness and an appropriate imputation model. Diagnostic checks will be performed to assess this.

#### Plans to give access to the full protocol, participant-level data, and statistical code

The full protocol is available to access from the authors. Once the main findings are published, the full anonymised participant-level dataset will be made available on request.

### Oversight and monitoring

#### Composition of the coordinating centre and trial steering committee

All day-to-day activities of the trial are co-ordinated at QMUL by the central study team who are Professor Stephanie Taylor (co-CI), the programme manager, Mr. Imran Khan, researchers Dr. Sheila Donovan and Dr Elisavet Moschopoulou, and the research administrator Ms. Shahd Mekki (previously Mr Colin Houlihan).

There are two committees and one patient and carer representatives’ group. The committees include the following: the programme management group (PMG) and the programme steering committee (PSC). The PMG includes the co-CIs, a range of clinical co-applicants, the central study team, site PIs and researchers, and patient and carer representatives. The PMG will meet regularly (at least every 2–3 months) to discuss the progress of the trial.

The PSC includes an independent chair, a clinician/epidemiologist, a researcher, and a patient representative. The non-independent members are the co-CIs. The progress of the trial will be monitored and supervised by the PSC. The PSC has been established in accordance with the Medical Research Council (MRC) guidelines [49].

The patient and carer representatives group include patients who are living with cancer or individuals who have had caring responsibilities for a person with cancer. The representatives have been actively involved in the preparation of the application, providing invaluable feedback. We have actively recruited members of the public who reflect the full demographic profile of the population our research is likely to impact, including those from seldom heard groups (e.g. members of diverse communities).

#### Composition of the data monitoring committee, its role and reporting structure

Due to the low-risk nature of the study, there will be no separate data monitoring committee (DMC). The PSC will encompass the role of a DMC.

#### Adverse event reporting and harms

We consider that the trial carries a very low risk of any adverse reactions (AR). Acceptance and commitment therapy is widely used today and is safe to use.

Expected adverse events (AE) include planned/elective hospitalisations or unplanned but expected hospitalisation due to cancer recurrence. These are expected during the trial and will not be collected.

The only AEs that we will collect are the following:

1. Known injuries as a result of exercise, if exercise support was used as part of the intervention delivery; and,
2. Suicidal ideation.

AEs will be logged on the Adverse Event Reporting Log available via the online portal. A copy of the log is also included in the investigator site file. The study team will be informed of the above listed AEs as soon as possible.

Should a participant become distressed about their situation and their condition or, more seriously, expresses suicidal intent or is at risk of harm to themselves or others, the therapists are fully trained and experienced to deal with such circumstances. These events will be reported to TC (lead therapist) who will decide on the appropriate action.

A severe adverse event (SAE) occurring to a research participant will be reported to the sponsor where in the opinion of the lead therapist the event was:

1. Related—that is, it resulted from administration of any research procedures; and,
2. Unexpected—that is the type of event is not considered as an expected occurrence (death of a participant due to cancer recurrence is expected and will not be reported to the sponsor).

The co-CI or sponsor will complete and send a SAE report to the REC within 15 days of becoming aware of the event. Recorded AEs and SAEs are summarised for the steering committee.

After a related or unexpected SAE (except for death), a decision will be made by the study team, after advice from the relevant authorities and the participant’s clinical team, as to whether the participant should be withdrawn from either their randomised treatment or from the trial. However, we do not envisage such a situation.

### Frequency and plans for auditing trial conduct

The study will be monitored and audited by the study sponsors Queen Mary University of London and Barts Health NHS Trust.

### Plans for communicating important protocol amendments to relevant parties

Amendments will be submitted to the sponsor for assessment, categorisation, and approval, prior to submission to the Health Research Authority and REC where necessary. The amendment history will be tracked via version and date control of the protocol and associated documents.

### Dissemination plans

Throughout the study, we will build interest in the study by distributing a regular electronic newsletter to all interested stakeholders. We will use social media, e.g. a dedicated twitter account to raise awareness of, and interest in, the project. The study will have a dedicated, up to date website (https://surecanstudy.qmul.ac.uk/).

We will submit abstracts for the main findings to be presented at scientific and health service-related conferences. The conference presentations will also aid the dissemination of our findings to clinicians, patients, and charities. Papers will be prepared and submitted in peer reviewed scientific journals with open access arrangements. We will approach a widely read, high-impact journal for the main trial paper.

We will work closely with our collaborator Macmillan Cancer Support to disseminate our results as widely as possible to patients and the public.

## Discussion

The common concerns for cancer survivors include fear of cancer recurrence, fatigue, body image concerns, lack of exercise, and unemployment in those of working age. These concerns may be associated with a poor quality of life (QoL). There is a clear need to support the increasing numbers of cancer survivors in the UK and beyond and a need for robust evidence-based studies assessing the effectiveness of aftercare available to them. The SURECAN study aims to address this need by identifying those cancer survivors with low QoL who are often receiving inadequate aftercare following their cancer treatment and offer them an intervention known as acceptance and commitment therapy (plus) (ACT+) to investigate if it improves their QoL. This intervention is suited to such patients to aid improvement in their health and well-being after cancer. To our knowledge, this is the first time an intervention based on ACT that integrates options for physical exercise and work/vocational activities support has been developed and evaluated in a large study for its effectiveness in addressing the specific needs and concerns of cancer survivors.

The COVID-19 pandemic caused much disruption to the running of cancer services and reduced communication and contact between cancer patients and their clinicians. SURECAN recruitment was therefore adapted to encompass recruiting patients either face-to-face in clinic or remotely (as described in the ‘Recruitment’ section). The intervention can be delivered online or over the phone, and training of therapists can also be delivered online.

This trial will provide us with evidence determining if there is an improvement in QoL while being cost-effective of ACT+ compared with usual aftercare. We will also determine if ACT+ reduces any anxiety and depression and helps participants manage the fear of the cancer coming back and if there is more engagement with activities that are meaningful to the participant.

### Trial status

Recruitment to the trial commenced in March 2021 and scheduled to end on 30 November 2023. Data collection will continue until 30 November 2024. The current version of the protocol is Version 5.0, 2 October 2023. The protocol amendments since commencement of the trial include the following: administrative updates, detailing the adaptations to the recruitment process during the COVID-19 pandemic, extending the recruitment period due to the pandemic, widening the eligibility criteria to include other tumour groups with good survival, addition of new sites, and other various minor changes to the layout and content of the protocol. All changes to the protocol have been approved by the South West - Cornwall & Plymouth Research Ethics Committee. The manuscript was submitted for publication 1 week prior to the recruitment end date. The submission has been delayed as parts of the trial procedures are needed to be adapted to recruit during and after the COVID-19 pandemic environment. Please refer to the ‘Discussion’ and ‘Recruitment’ sections.

## Supplementary Information

**Additional file 1.**

**Additional file 2: Table S1.** All items from the World Health Organization Trial Registration Data Set for this protocol.

## Data Availability

The final trial dataset will be available upon request to the corresponding authors once all analysis is complete.

## Abbreviations

ACT+: Acceptance and commitment therapy enhanced by exercise therapy and vocational rehabilitation, as appropriate
AE: Adverse event
AR: Adverse reaction
CI: Chief investigator
CRF: Case report form
DMC: Data monitoring committee
FACT-G: Functional Assessment of Cancer Therapy – General
GDPR: General Data Protection Regulation
IAPT: Improving Access to Psychological Therapies
ICF: Informed consent form
MRC: Medical Research Council
PCTU: Pragmatic Clinical Trials Unit, Queen Mary University of London
PI: Principal investigator
PIS: Participant information sheet
PMG: Programme management group
PSC: Programme steering committee
QMUL: Queen Mary University of London
QoL: Quality of life
QALY: Quality-adjusted life years
RCT: Randomised controlled trial
REC: Research ethics committee
SAE: Serious adverse event
SOP: Standard operating procedure
SURECAN: SUrvivors Rehabilitation Evaluation after CANcer
