# Supplementary material for: Study protocol for a pragmatic randomised controlled trial of comparing enhanced acceptance and commitment therapy plus (+) added to usual aftercare versus usual aftercare only, in patients living with or beyond cancer: SUrvivors’ Rehabilitation Evaluation after CANcer (SURECAN) trial
Source: Trials. 2024 Apr 2;25:228. doi: 10.1186/s13063-024-08062-4 (PMC10985882; doi:10.1186/s13063-024-08062-4)
Supplement: Supplementary file 2 — Additional file 2: Table S1. All items from the World Health Organization Trial Registration Data Set for this protocol. [file 13063_2024_8062_MOESM2_ESM.docx]

**Table S1. All items from the World Health Organization Trial Registration Data Set for this protocol**

| **Date Category** | **Information** |
| --- | --- |
| Primary registry and trial identifying number | ISRCTN67900293 |
| Date of registration in primary registry | 9 December 2019 |
| Funder name & reference | National Institute of Health Research (NIHR) Programme Grant for Applied Research (PGfAR): RP-PG-0616-20002 |
| Public title: | SUrvivors Rehabilitation Evaluation after CANcer (SURECAN) randomised control trial |
| Scientific title | A pragmatic randomised controlled trial of comparing enhanced Acceptance and Commitment Therapy Plus (+) added to usual aftercare versus usual aftercare only, in patients living with or beyond cancer: SUrvivors' Rehabilitation Evaluation after CANcer (SURECAN) trial. |
| Countries of recruitment | England |
| Health condition(s) or problem(s) studied | Cancer; mental health |
| Intervention | ACT+ delivered by NHS Talking Therapies, specialist services, and cancer charities. The intervention consists of up to eight sessions at weekly or fortnightly intervals using different modalities of delivery to suit individual needs i.e., face-to-face sessions, over the phone or skype. |
| Inclusion criteria | Patients within 24 months of having completed cancer treatment of the index cancer, (or about to complete) with curative intent / long term remission for: breast cancer, lower gastrointestinal cancer, a urological cancer, a haematological cancer, head and neck cancer, and any other common cancer with good survival.  Aged 18 years or over  Ability to give informed consent  Sufficient fluency in spoken English to be able to participate in a talking-based therapy delivered in English  With a score of 78 or less on the Functional Assessment of Cancer Therapy – General (FACT-G) |
| Exclusion criteria | Will not have not completed their cancer treatment by the commencement of the trial (excepting those receiving long-term, ongoing maintenance treatment e.g. androgen suppression therapy in prostate cancer).  Receiving treatment for symptom control alone  Currently receiving another psychological intervention (NB participants taking antidepressants or anxiolytic drugs remain eligible)  Other serious co-morbid condition which would make it difficult for the participant to receive a talking-based one-to-one intervention  Require urgent psychiatric or clinical psychology assessment |
| Study type | Multi-centre, pragmatic, theory driven, randomised controlled trial |
| Date of first enrolment | 22 March 2021 |
| Target sample size | 344 |
| Recruitment status | Ended recruitment |
| Definitive trial outcomes | *Primary outcomes:*  Functional Assessment of Cancer Therapy: General scale (FACT-G) at 12 months  Primary health economics outcome: Quality adjusted life years based on EQ-5D-5L and net monetary benefit at 12 months  *Secondary outcomes:*  FACT-G sub-scale scores  Positive and negative Impact of Cancer scales  Fear of cancer recurrence inventory  Hospital Anxiety and Depression scale  Chalder Fatigue questionnaire  Physical activity |
